# Comparative evaluation of TACE-RFA versus monotherapies on a background of systemic therapy for recurrent hepatocellular carcinoma: a propensity score-matched analysis

**DOI:** 10.3389/fonc.2026.1825546

**Published:** 2026-08-26

**Authors:** Wendi Zhang, Zuhao Ye, Jinfa Wang, Xia Ren, Liping Ying, Yifeng Wu, Zongyang Wu, Chang Xin, Jiye Feng

**Affiliations:** 1 Department of Hepatobiliary and Pancreatic Surgery, The Affiliated People’s Hospital of Ningbo University, Ningbo, Zhejiang, China; 2 Ningbo University Health Science Center, Ningbo, China

**Keywords:** cost-effectiveness, progression-free survival, propensity score matching, radiofrequency ablation, recurrent hepatocellular carcinoma, transarterial chemoembolization

## Abstract

**Background:**

The optimal locoregional treatment for recurrent hepatocellular carcinoma (rHCC) in the era of potent systemic therapy remains undefined. This study aimed to compare the efficacy, safety, and resource consumption of transarterial chemoembolization combined with radiofrequency ablation (TACE-RFA) versus TACE or RFA monotherapy, specifically when administered on a uniform background of systemic therapy (Lenvatinib plus PD-1 inhibitors).

**Methods:**

We retrospectively reviewed 243 patients with rHCC who met strict eligibility criteria (tumor size 3.0–7.0 cm, ≤3 recurrent nodules, and preserved liver function, corresponding to Barcelona Clinic Liver Cancer (BCLC) A/B and China Liver Cancer (CNLC) Ia–IIa stages. Crucially, all included patients uniformly received standardized peri-procedural systemic therapy (Lenvatinib and Tislelizumab). Based on the actual locoregional strategies, the cohort was divided into TACE-RFA (n=56), TACE alone (n=114), or RFA alone (n=73) groups. To mitigate selection bias, 1:1 propensity score matching (PSM) was employed, generating two matched cohorts: Combination vs. TACE alone (54 pairs) and Combination vs. RFA alone (54 pairs). Survival outcomes, post-procedural pain, hospital stay, and costs were evaluated.

**Results:**

After PSM, baseline characteristics were well-balanced. The median progression-free survival (PFS) in the combination group was 21.4 months, significantly superior to the matched TACE monotherapy cohort (13.5 months, P < 0.001) and the independently matched RFA monotherapy cohort (10.4 months, P < 0.001). Overall survival (OS) showed no significant differences among the groups (median OS 48.9–50.5 months, all P > 0.05), likely because the uniform administration of potent systemic therapy drastically prolonged the baseline survival for all patients, thereby diluting the OS advantage of local control. Safety profiles were comparable across all modalities (P > 0.05). Predictably, combination therapy incurred longer median hospital stays and higher total costs compared to monotherapies (all P < 0.001).

**Conclusion:**

For rHCC patients receiving concurrent Lenvatinib and PD-1 inhibitors, sequential TACE-RFA therapy provides a profound and independent progression-free survival benefit over locoregional monotherapies without increasing patient discomfort. The superior local tumor control justifies the anticipated initial increases in perioperative resource consumption.

## 1 Introduction

Hepatocellular carcinoma (HCC) ranks as the sixth most common malignancy and the third leading cause of cancer-related mortality worldwide (1, 2). Despite advances in curative treatments such as hepatic resection and liver transplantation, the long-term prognosis remains daunting due to the high frequency of recurrence, which can reach up to 70% within five years after surgery (3). Recurrent HCC (rHCC) poses a distinct clinical challenge compared to primary HCC, as treatment options are often restricted by limited liver functional reserve and the complex anatomical location of recurrent lesions. Consequently, repeated locoregional therapies, rather than re-resection, have become the mainstay of management for these patients.

Currently, Radiofrequency Ablation (RFA) and Transarterial Chemoembolization (TACE) are the standard modalities for rHCC. However, each monotherapy has inherent limitations. RFA efficacy is often compromised by the “heat sink effect” in perivascular tumors and the risk of incomplete ablation in lesions larger than 3 cm (4). Conversely, TACE rarely achieves complete tumor necrosis as a standalone therapy, leading to high local recurrence rates (5). To overcome these limitations, the combination of TACE and RFA has been proposed as a synergistic strategy (6). Theoretically, TACE creates an ischemic environment by occluding tumor feeding arteries, which minimizes heat loss during subsequent ablation and facilitates a larger ablation volume. Furthermore, the chemotherapeutic agents delivered by TACE can target peripheral microsatellites that might be missed by RFA alone.

While accumulating evidence supports the efficacy of transarterial chemoembolization combined with radiofrequency ablation (TACE-RFA) combination therapy for HCC, emerging data specifically addressing recurrent HCC remain relatively limited and have yielded heterogeneous conclusions (7, 8). However, the addition of TACE to RFA requires an additional invasive procedure and may increase procedural complexity, treatment burden, hospitalization requirements, and healthcare resource utilization. In the era of value-based healthcare, it is crucial to determine whether the potential survival advantage justifies the additional economic burden and safety risks. Furthermore, the therapeutic landscape of HCC has been substantially reshaped by targeted agents and immune checkpoint inhibitors, and increasing attention has shifted toward integrating locoregional and systemic therapies rather than considering them as isolated treatment modalities (9, 10). However, few studies have rigorously evaluated this trade-off using real-world data, and most existing comparisons are limited by significant baseline imbalances.

Therefore, this study aimed to provide a comprehensive evaluation of TACE-RFA combination therapy versus TACE or RFA monotherapy for patients with recurrent HCC, specifically focusing on a real-world cohort uniformly receiving a consistent systemic-treatment backbone of Lenvatinib plus programmed cell death protein 1 (PD-1) inhibitors. To mitigate the selection bias common in observational studies, we utilized Propensity Score Matching (PSM) to rigorously balance baseline characteristics—including tumor burden and liver function—we sought to compare not only the long-term efficacy (Progression-Free Survival [PFS] and Overall Survival[OS]) but also the safety profile and resource consumption (hospital stay and total costs), thereby offering rigorous evidence to guide clinical decision-making in the modern era of combined modalities.

## 2 Methods

### 2.1 Patients and study design

This retrospective cohort study reviewed the medical records of patients diagnosed with recurrent hepatocellular carcinoma (rHCC) at The Affiliated People’s Hospital of Ningbo University between January 2019 and October 2024. Initially, a total of 403 patients who experienced HCC recurrence after initial hepatectomy were screened for eligibility.

The inclusion criteria were strictly defined as follows: ① diagnosis of rHCC confirmed by histopathology or clinical criteria (combining imaging, tumor markers, and clinical history); ② maximum diameter of recurrent tumors ranging from 3.0 to 7.0 cm, with the number of recurrent nodules ≤ 3. This precisely corresponds to specific subsets of the early (Stage A) and intermediate (Stage B) stages according to the 2026 updated Barcelona Clinic Liver Cancer (BCLC) staging system (11), as well as stages Ia, Ib, and IIa in the 2024 China liver cancer staging (CNLC) guidelines (12) ;③ preserved liver function of Child-Pugh class A or well-compensated class B (strictly defined as a score of 7, without refractory ascites or recent clinical decompensation); ④ Eastern Cooperative Oncology Group performance status (ECOG PS) score of 0; and ⑤ patients who received TACE alone, RFA alone, or TACE combined with RFA for the treatment of recurrent lesions.

The exclusion criteria were as follows: ① presence of macroscopic vascular invasion (e.g., portal vein tumor thrombosis or hepatic vein thrombosis) or extrahepatic metastasis (e.g., lung, bone, or lymph node metastasis); ② history of liver transplantation or presence of other malignancies within the past 5 years; ③ severe comorbidities including severe cardiopulmonary or renal dysfunction precluding treatment tolerance, active infections, or sepsis; ④ incomplete clinical data (e.g., lack of contrast-enhanced computed tomography (CT) or magnetic resonance imaging (MRI) within 1 month prior to treatment) or lost to follow-up (survival status unattainable) and ⑤ discontinuation of the standardized systemic therapy (Lenvatinib plus Tislelizumab) prior to documented disease progression due to immune-related adverse events, drug intolerance, or early resistance.

**Figure 1:**
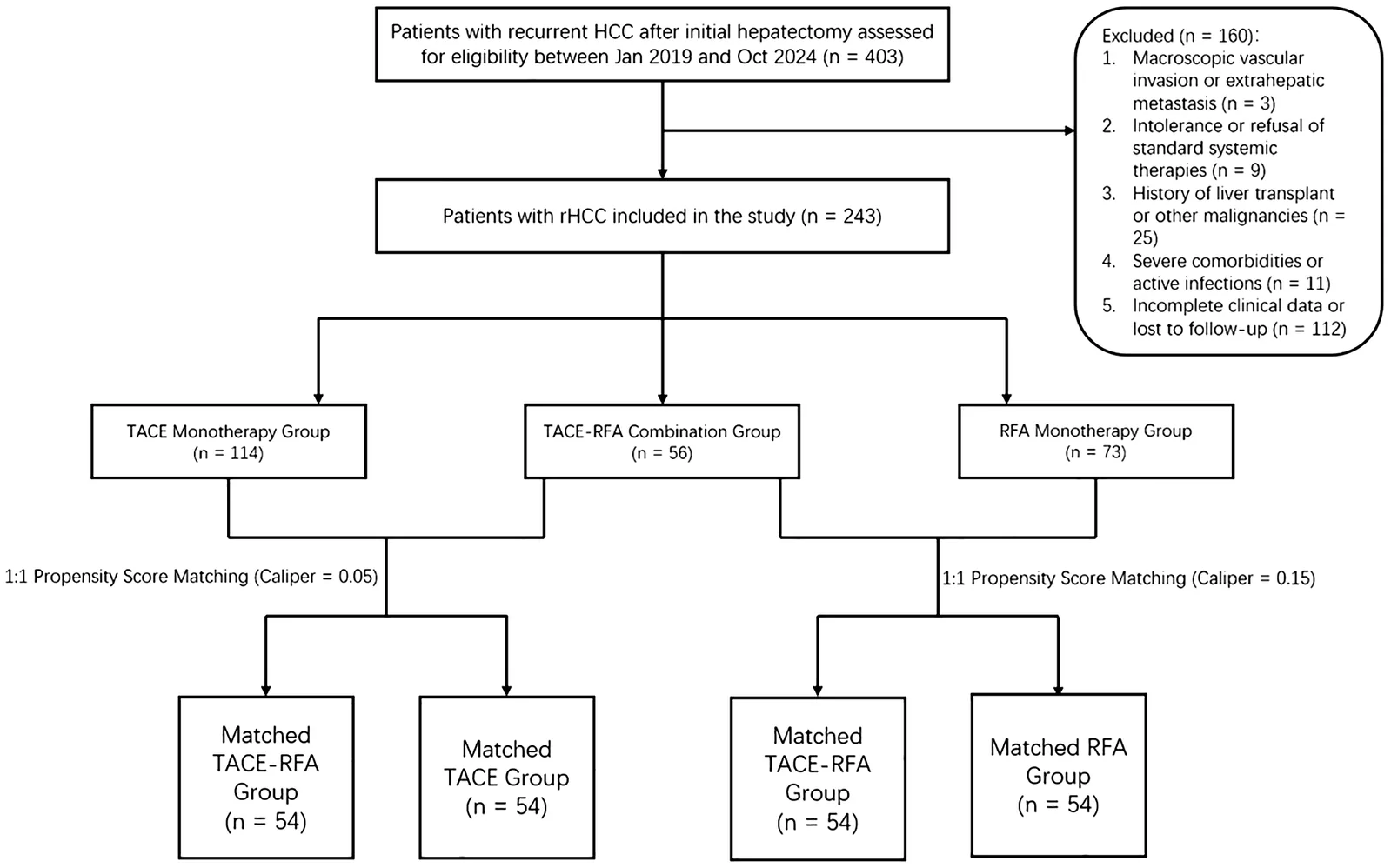
Study flowchart of patient selection and propensity score matching.

After rigorous screening, 243 patients met the criteria and were ultimately included in the study (**Figure 1**). Based on the actual locoregional treatments received, these patients were categorized into three cohorts: the TACE-RFA combination group (n = 56), the TACE monotherapy group (n = 114), and the RFA monotherapy group (n = 73).

The study protocol was approved by the Ethics Committee of The Affiliated People’s Hospital of Ningbo University. Given the retrospective nature of the study, the requirement for written informed consent was waived by the institutional review board. All patient data were analyzed anonymously in compliance with the Declaration of Helsinki.

### 2.2 Treatment procedures

#### 2.2.1 Standard TACE and RFA techniques

Transarterial chemoembolization (TACE) was performed using the conventional Seldinger technique in adherence to the Cardiovascular and Interventional Radiological Society of Europe (CIRSE) and Initiative on Superselective Conventional Transarterial Chemoembolization Results (INSPIRE) standards of practice (13, 14). Briefly, following the introduction of a catheter via the femoral artery, intraoperative digital subtraction angiography (DSA) and Cone-Beam CT (CBCT) were routinely utilized to meticulously identify all tumor-feeding arteries and accurately assess the intended embolization range. A microcatheter—commonly utilizing 1.98Fr, 2.4Fr, or 2.8Fr systems (e.g., Masters Parkway Soft [Asahi Intecc, Pathumthani, Thailand], Direxion [Boston Scientific, Marlborough, MA, USA], Progreat CC-MC913SN [Terumo Corporation, Kakamigahara, Gifu, Japan], or Maestro [Merit Medical Systems, South Jordan, UT, USA])—was systematically employed. The procedural objective was to achieve refined, superselective catheterization of the specific tumor-feeding branches; in cases where superselective catheterization was technically unattainable, CBCT-guided regional embolization of the corresponding hepatic segment was performed. A well-mixed water-in-oil emulsion was prepared by first dissolving 40 mg of epirubicin in 3–4 mL of normal saline, and then thoroughly emulsifying it with 6–8 mL of Lipiodol (titrated to tumor size) using a 3-way stopcock connecting two syringes. This emulsion was slowly injected into the target vessels. Crucially, to achieve complete arterial stasis, prevent rapid washout, and ensure durable flow reduction, supplemental embolic agents—specifically blank microspheres (300–500 μm) or absorbable gelatin sponge particles (100–350 μm)—were administered following the emulsion injection.

Radiofrequency ablation (RFA) was conducted percutaneously under real-time ultrasound or CT guidance using theCool-tip RF System(Radionics, Burlington, MA, USA) and appropriate electrodes, in adherence to international consensus guidelines for thermal ablation (14). The ablation parameters were tailored according to tumor size and location. For intermediate-to-large recurrent tumors (>3 cm), overlapping multi-station ablations were systematically applied to ensure complete tumor coverage. The overarching goal was to encompass the entire target lesion with a continuous circumferential safety margin of 0.5 to 1.0 cm, a strategy strongly recommended to minimize local tumor progression (15). Completeness of the ablation margin was routinely assessed via contrast-enhanced CT or MRI performed 1 month post-procedure.

#### 2.2.2 Group-specific treatment protocols

In this retrospective study, treatment group assignment was determined by the actual completed locoregional strategy (an “as-treated” approach). Patients initially intended for combination therapy who failed to proceed to RFA due to clinical deterioration or disease progression during the interval were not included in the finalized combination cohort.

For patients in the TACE-RFA combination cohort, a sequential treatment strategy was employed, wherein percutaneous RFA was planned within 4 weeks following the initial TACE procedure. In contrast, individuals assigned to the TACE monotherapy group received TACE alone. Following their initial procedure, contrast-enhanced imaging was evaluated every 4 to 8 weeks. An “on-demand” repeated TACE strategy was adopted: if residual viable tumor tissue (indicating incomplete initial response) was detected, repeated TACE was administered—provided liver function permitted—until complete tumor necrosis was achieved or intolerable toxicities occurred. Crucially, to rigorously define survival endpoints, any new local recurrence or distant metastasis detected after achieving an initial complete response was explicitly recorded as a PFS event, rather than an indication for continuing the initial treatment course.

Finally, patients in the RFA monotherapy group underwent RFA alone. Routine contrast-enhanced imaging was assessed 1 month post-procedure. If incomplete ablation was identified, a supplementary ablation session was promptly executed to ensure complete tumor clearance.

The actual time interval between sequential procedures and the total number of treatment sessions required to achieve the initial therapeutic goal were meticulously recorded for all patients.

#### 2.2.3 Standard systemic background therapy and clinical pathway

All patients included in this study followed a uniform clinical trajectory: initial curative hepatectomy, regular surveillance (imaging and tumor markers, alongside continuous antiviral therapy for hepatitis B virus (HBV)- or hepatitis C virus (HCV)-positive patients), and subsequent locoregional treatment upon the diagnosis of recurrence.

Reflecting contemporary multidisciplinary management paradigms, all cohorts uniformly received a standardized systemic therapy regimen during the peri-procedural period of their recurrence. Specifically, Lenvatinib was administered orally at a dose of 8 mg once daily, and Tislelizumab (a PD-1 inhibitor) was administered intravenously at a dose of 200 mg every 21 days. As defined in our strict exclusion criteria, all analyzed patients maintained this continuous and uniform systemic therapy until the next disease progression. Consequently, the adherence rate in our finalized cohort is effectively uniform, ensuring that the observed survival differences were primarily driven by the variations in locoregional strategies rather than systemic confounding factors.

### 2.3 Follow-up and endpoints

Following the completion of the designated locoregional treatments, all patients were subjected to a standardized follow-up protocol. Routine follow-up visits were scheduled every 3 to 6 months. During these visits, clinical assessments, routine laboratory tests (including liver function and complete blood count), and serum tumor markers (such as alpha-fetoprotein [AFP] and protein induced by vitamin K absence or antagonist-II [PIVKA-II]) were evaluated. Contrast-enhanced computed tomography (CT) or magnetic resonance imaging (MRI) of the abdomen was routinely performed every 6 to 12 months, or immediately if disease progression was clinically suspected based on elevated tumor markers or deteriorating symptoms.

The primary endpoint of this study was progression-free survival (PFS), defined as the time interval from the initiation of the locoregional treatment for recurrent HCC to the date of the first documented disease progression or death from any cause. Disease progression—encompassing local tumor progression, new intrahepatic lesions, or extrahepatic metastasis—was radiologically assessed according to the modified Response Evaluation Criteria in Solid Tumors (mRECIST) (16). To further evaluate the efficacy of local control, the patterns of first disease progression were meticulously recorded and categorized into two main patterns: local tumor progression (LTP, defined as the appearance of viable tumor tissue at the margin of the previously treated lesion), and distant progression (which encompasses new intrahepatic lesions and extrahepatic metastasis).The secondary endpoint was overall survival (OS), calculated from the date of the initial recurrent treatment to the date of death from any cause or the last follow-up. Median follow-up time was calculated using the reverse Kaplan-Meier method. Patients who remained alive without disease progression at the end of the study period, or those who were lost to follow-up, were censored at the date of their last clinical contact. The follow-up cutoff date was December 31, 2025.

### 2.4 Statistical analysis

Continuous variables were expressed as the mean ± standard deviation (SD) or median with interquartile range (IQR), depending on the normality of the distribution, and compared using the independent samples t-test or Mann-Whitney U test. Categorical variables were presented as frequencies and percentages, and comparisons between groups were performed using the Chi-square test or Fisher’s exact test, as appropriate.

To minimize potential selection bias and control for baseline confounders inherent in the retrospective study design, Propensity Score Matching (PSM) was implemented (17). Propensity scores were estimated using a multivariable logistic regression model based on the following eight clinically significant covariates: age, sex, tumor size, tumor number, alpha-fetoprotein (AFP) level, PIVKA-II, etiology (hepatitis B/C or others), and Child-Pugh grade. Two separate 1:1 matching procedures were performed using a nearest-neighbor matching algorithm without replacement. First, patients in the TACE-RFA combination group were matched with those in the TACE monotherapy group with a strict caliper width set at 0.05 of the standard deviation of the logit of the propensity score. Second, a separate matching was conducted between the TACE-RFA combination group and the RFA monotherapy group utilizing a caliper width of 0.15. The balance of baseline characteristics before and after matching was strictly evaluated, including variables not directly incorporated in the matching models (e.g., platelet count, albumin, and prothrombin time), ensuring optimal group comparability.

Survival curves for progression-free survival (PFS) and overall survival (OS) were generated using the Kaplan-Meier method, and differences between the matched groups were compared using the stratified log-rank test. Furthermore, subgroup analyses were strictly performed for the primary endpoint (PFS) by stratifying patients according to predefined tumor size categories (3.0–5.0 cm and 5.1–7.0 cm). To identify independent prognostic factors, variables with a P value < 0.05 in the univariate analysis, along with the treatment modality, were incorporated into a multivariate Cox proportional hazards regression model to calculate the hazard ratios (HRs) and 95% confidence intervals (CIs).

All statistical analyses were performed using SPSS software (version18.0). A two-sided P value of < 0.05 was considered statistically significant.

## 3 Results

### 3.1 Baseline characteristics before and after PSM

A total of 243 patients with recurrent hepatocellular carcinoma (rHCC) who met the inclusion criteria were included in this study. Based on the initial locoregional treatments received, the cohort was divided into the TACE-RFA combination group (n = 56), the TACE monotherapy group (n = 114), and the RFA monotherapy group (n = 73). Before propensity score matching, several baseline clinical and tumor characteristics exhibited significant imbalances between the combination group and the two monotherapy groups, justifying the necessity of statistical adjustment. In the entire cohort receiving combination therapy (n = 56), the actual time interval between the initial TACE procedure and the subsequent RFA treatment was meticulously recorded. The median TACE-to-RFA interval was remarkably short at 5.0 days (interquartile range [IQR], 4.0–7.8 days; range, 2–21 days). No patients intended for combination therapy failed to complete the sequential RFA due to interval clinical deterioration.

Regarding the initial procedural burden to achieve complete tumor clearance, the vast majority of patients in the monotherapy groups required only a single treatment session. Specifically, in the TACE monotherapy group (n=114 before PSM), 12 patients (10.5%) required a second “on-demand” TACE session to achieve complete necrosis, with a median interval of 35.5 days (range, 29–44 days) between the two sessions. In the RFA monotherapy group (n=73 before PSM), only 4 patients (5.5%) required a supplementary ablation, with a median interval of 34.5 days (range, 30–37 days). No patient in any group required more than two sessions for the initial treatment course.

#### 3.1.1 Combination vs TACE monotherapy

To compare the combination therapy with TACE alone, a 1:1 PSM was performed (caliper width = 0.05). This yielded 54 strictly matched pairs (n = 108). After matching, all baseline characteristics—including demographics, tumor burden (size and number), clinical staging (BCLC and CNLC stages), detailed liver function profiles (such as exact Child-Pugh score, albumin-bilirubin (ALBI) grade, platelet count, and albumin), and recurrence-free interval—were well-balanced between the two groups, with no statistically significant differences observed (all P > 0.05).

#### 3.1.2 Combination vs RFA monotherapy

Similarly, a separate 1:1 PSM (caliper width = 0.15) was conducted to compare the combination therapy with RFA alone, resulting in 54 matched pairs (n = 108). Following this matching procedure, all analyzed covariates were optimally balanced between the combination and RFA groups, demonstrating excellent comparability (all P > 0.05).

The detailed baseline demographic and clinical characteristics of the study population before and after the two respective matching procedures are comprehensively summarized in **Table 1** and **Table 2**.

**Table 1:** Baseline demographic and clinical characteristics of patients before and after propensity score matching (Combination vs. TACE alone).

Variables	Before PSM			After PSM		
	Combined (n=56)	TACE (n=114)	*P* value	Combined (n=54)	TACE (n=54)	*P* value
Age (years)	62.2 ± 11.0	63.1 ± 8.9	0.602	62.6 ± 10.9	62.7 ± 8.2	0.937
Sex (Male/Female)	48 (85.7)/8 (14.3)	100 (87.7)/14 (12.3)	0.714	47 (87.0)/7 (13.0)	47 (87.0)/7 (13.0)	1.000
ALT(U/L), median (IQR)	23.00 (13.25, 29.75)	24.00 (17.00, 34.25)	0.130	23.0 (13.0–29.3)	23.0 (15.0–31.3)	0.742
AST (U/L), median (IQR)	26.00 (21.00, 32.75)	26.00 (18.00, 32.00)	0.332	26.0 (21.0–32.3)	25.0 (17.8–30.3)	0.167
TBIL (μmol/L), median (IQR)	15.85 (10.80, 20.70)	13.40 (9.40, 18.93)	0.121	16.1 (10.8–20.8)	13.5 (8.9–19.0)	0.166
ALB (g/L), median (IQR)	42.35 (37.65, 45.13)	38.85 (34.00, 43.20)	0.005*	42.4 (37.3–45.2)	40.9 (34.9–43.9)	0.068
PT (sec), median (IQR)	11.25 (10.90, 12.28)	11.35 (10.90, 11.93)	0.855	11.3 (10.9–12.4)	11.2 (10.8–11.7)	0.297
INR, median (IQR)	0.99 (0.96, 1.03)	1.01 (0.97, 1.06)	0.544	0.99 (0.96–1.04)	0.99 (0.96–1.03)	0.609
PLT (10^9^/L), median (IQR)	118.00 (98.00, 150.25)	108.50 (93.00, 123.00)	0.086	119.0 (98.0–151.5)	113.5 (97.8–156.5)	0.839
AFP (ng/mL), median (IQR)	7.92 (3.52, 106.85)	5.44 (3.15, 64.76)	0.307	7.9 (3.4–104.8)	5.8 (3.1–135.9)	0.553
PIVKA-II (mAU/mL), median (IQR)	60.95 (24.68, 345.53)	50.79 (21.71, 844.03)	0.451	72.1 (24.8–397.0)	57.0 (22.0–408.6)	0.457
Tumor Size (cm), median (IQR)	4.05 (3.50, 5.20)	5.10 (4.08, 6.00)	<0.001*	4.2 (3.5–5.2)	4.2 (3.6–5.4)	0.608
Etiology (HBV/Others)	54 (96.4)/2 (3.6)	105 (92.1)/9 (7.9)	0.343	51 (94.4)/3 (5.6)	52 (96.3)/2 (3.7)	1.000
Antiviral Therapy (Yes/No)	52 (92.9)/4 (7.1)	106 (93.0)/8 (7.0)	1.000	51 (94.4)/3 (5.6)	50 (92.6)/4 (7.4)	1.000
Tumor Number (1/2/3)	26 (46.4)/14 (25.0)/16 (28.6)	28 (24.6)/49 (43.0)/37 (32.5)	0.011*	23 (42.6)/18 (33.3)/13 (24.1)	24 (44.4)/14 (25.9)/16 (29.6)	0.660
Child-Pugh class (A/B)	52 (92.9)/4 (7.1)	107 (93.9)/7 (6.1)	0.753	50 (92.6)/4 (7.4)	49 (90.7)/5 (9.3)	1.000
Exact Child-Pugh score (A5/A6/B7)	46 (82.1)/6 (10.7)/4 (7.1)	79 (69.3)/28 (24.6)/7 (6.1)	0.105	44 (81.5)/6 (11.1)/4 (7.4)	39 (72.2)/10 (18.5)/5 (9.3)	0.504
ALBI grade (1/2/3)	37 (66.1)/18 (32.1)/1 (1.8)	56 (49.1)/58 (50.9)/0 (0.0)	0.019*	36 (66.7)/17 (31.5)/1 (1.9)	32 (59.3)/22 (40.7)/0 (0.0)	0.426
BCLC stage (A/B)	28 (50.0)/28 (50.0)	30 (26.3)/84 (73.7)	0.002*	26 (48.1)/28 (51.9)	24 (44.4)/30 (55.6)	0.700
CNLC stage (Ia/Ib/IIa)	20 (35.7)/8 (14.3)/28 (50.0)	16 (14.0)/14 (12.3)/84 (73.7)	0.003*	18 (33.3)/8 (14.8)/28 (51.9)	15 (27.8)/9 (16.7)/30 (55.6)	0.819
Recurrence-free interval (months), median (IQR)	34.7 (25.3–44.5)	33.9 (27.8–44.5)	0.435	35.3 (25.3–45.2)	36.6 (28.5–44.8)	0.312

TACE, transarterial chemoembolization; RFA, radiofrequency ablation; PSM, propensity score matching; ALT, alanine aminotransferase; AST, aspartate aminotransferase; TBIL, total bilirubin; ALB, albumin; PT, prothrombin time; INR, international normalized ratio; PLT, platelet count; AFP, alpha-fetoprotein; PIVKA-II, protein induced by vitamin K absence or antagonist-II; HBV, hepatitis B virus; ALBI, albumin-bilirubin; BCLC, Barcelona Clinic Liver Cancer; CNLC, China Liver Cancer; IQR, interquartile range. Continuous variables are expressed as mean ± standard deviation or median (IQR). Categorical variables are expressed as number (percentage). *P < 0.05 indicates statistical significance.

**Table 2:** Baseline demographic and clinical characteristics of patients before and after propensity score matching (Combination vs. RFA alone).

Variables	Before PSM			After PSM		
	Combined (n=56)	RFA (n=73)	*P* value	Combined (n=54)	RFA (n=54)	*P* value
Age (years)	62.2 ± 11.0	64.9 ± 11.9	0.200	62.7 ± 10.4	64.8 ± 11.1	0.298
Sex (Male/Female)	48 (85.7)/8 (14.3)	55 (75.3)/18 (24.7)	0.146	46 (85.2)/8 (14.8)	44 (81.5)/10 (18.5)	0.606
ALT (U/L), median (IQR)	23.00 (13.25, 29.75)	20.00 (15.00, 24.50)	0.277	23.00 (13.75, 30.25)	22.00 (16.75, 28.00)	0.971
AST (U/L), median (IQR)	26.00 (21.00, 32.75)	22.00 (17.50, 31.00)	0.042*	26.00 (21.00, 33.00)	24.50 (19.00, 33.00)	0.534
TBIL (μmol/L), median (IQR)	15.85 (10.80, 20.70)	13.90 (9.50, 17.75)	0.116	15.45 (10.75, 20.50)	13.95 (9.60, 18.20)	0.289
ALB (g/L), median (IQR)	42.35 (37.65, 45.13)	40.50 (37.80, 44.05)	0.353	42.35 (37.40, 44.98)	40.45 (37.80, 43.70)	0.312
PT (sec), median (IQR)	11.25 (10.90, 12.28)	11.20 (10.95, 11.85)	0.527	11.30 (10.90, 12.38)	11.15 (10.78, 11.73)	0.181
INR, median (IQR)	0.99 (0.96, 1.03)	1.01 (0.99, 1.06)	0.164	1.00 (0.96, 1.04)	1.00 (0.97, 1.06)	0.518
PLT (10^9^/L), median (IQR)	118.00 (98.00, 150.25)	116.00 (101.00, 176.00)	0.709	118.00 (98.00, 151.50)	117.00 (100.50, 182.50)	0.574
AFP (ng/mL), median (IQR)	7.92 (3.52, 106.85)	5.12 (3.29, 22.84)	0.161	7.92 (3.61, 104.75)	4.89 (3.30, 17.48)	0.098
PIVKA-II (mAU/mL), median (IQR)	60.95 (24.68, 345.53)	30.52 (20.79, 143.81)	0.080	53.44 (24.46, 288.52)	29.58 (20.37, 217.34)	0.126
Tumor Size (cm), median (IQR)	4.05 (3.50, 5.20)	4.90 (3.85, 5.90)	0.003*	4.15 (3.50, 5.23)	4.70 (3.78, 5.50)	0.085
Etiology (HBV/Others)	54 (96.4)/2 (3.6)	59 (80.8)/14 (19.2)	0.008*	52 (96.3)/2 (3.7)	49 (90.7)/5 (9.3)	0.437
Antiviral Therapy (Yes/No)	52 (92.9)/4 (7.1)	60 (82.2)/13 (17.8)	0.076	50 (92.6)/4 (7.4)	49 (90.7)/5 (9.3)	1.000
Tumor Number (1/2/3)	26 (46.4)/14 (25.0)/16 (28.6)	28 (38.4)/23 (31.5)/22 (30.1)	0.610	26 (48.1)/13 (24.1)/15 (27.8)	21 (38.9)/17 (31.5)/16 (29.6)	0.578
Child-Pugh class (A/B)	52 (92.9)/4 (7.1)	70 (95.9)/3 (4.1)	0.467	51 (94.4)/3 (5.6)	51 (94.4)/3 (5.6)	1.000
Exact Child-Pugh score (A5/A6/B7)	46 (82.1)/6 (10.7)/4 (7.1)	59 (80.8)/11 (15.1)/3 (4.1)	0.603	45 (83.3)/6 (11.1)/3 (5.6)	43 (79.6)/8 (14.8)/3 (5.6)	0.927
ALBI grade (1/2/3)	37 (66.1)/18 (32.1)/1 (1.8)	47 (64.4)/25 (34.2)/1 (1.4)	0.928	36 (66.7)/17 (31.5)/1 (1.9)	35 (64.8)/18 (33.3)/1 (1.9)	1.000
BCLC stage (A/B)	28 (50.0)/28 (50.0)	28 (38.4)/45 (61.6)	0.186	28 (51.9)/26 (48.1)	21 (38.9)/33 (61.1)	0.176
CNLC stage (Ia/Ib/IIa)	20 (35.7)/8 (14.3)/28 (50.0)	11 (15.1)/17 (23.3)/45 (61.6)	0.021*	20 (37.0)/8 (14.8)/26 (48.1)	9 (16.7)/12 (22.2)/33 (61.1)	0.055
Recurrence-free interval (months), median (IQR)	34.7 (25.3–44.5)	29.9 (22.1–39.5)	0.240	35.3 (25.3–45.2)	30.1 (22.8–40.6)	0.267

TACE, transarterial chemoembolization; RFA, radiofrequency ablation; PSM, propensity score matching; ALT, alanine aminotransferase; AST, aspartate aminotransferase; TBIL, total bilirubin; ALB, albumin; PT, prothrombin time; INR, international normalized ratio; PLT, platelet count; AFP, alpha-fetoprotein; PIVKA-II, protein induced by vitamin K absence or antagonist-II; HBV, hepatitis B virus; ALBI, albumin-bilirubin; BCLC, Barcelona Clinic Liver Cancer; CNLC, China Liver Cancer; IQR, interquartile range. Continuous variables are expressed as mean ± standard deviation or median (IQR). Categorical variables are expressed as number (percentage). *P < 0.05 indicates statistical significance.

### 3.2 Survival outcomes

The median follow-up time for the entire cohort was 48.6 months (95% CI: 44.6–52.6 months). Following rigorous propensity score matching, the median follow-up times for the respective matched cohorts remained sufficiently long to reliably capture survival events: 46.9 months for the TACE-RFA versus TACE matched cohort, and 46.1 months for the TACE-RFA versus RFA matched cohort.

#### 3.2.1 Progression-free survival

**Figure 2:**
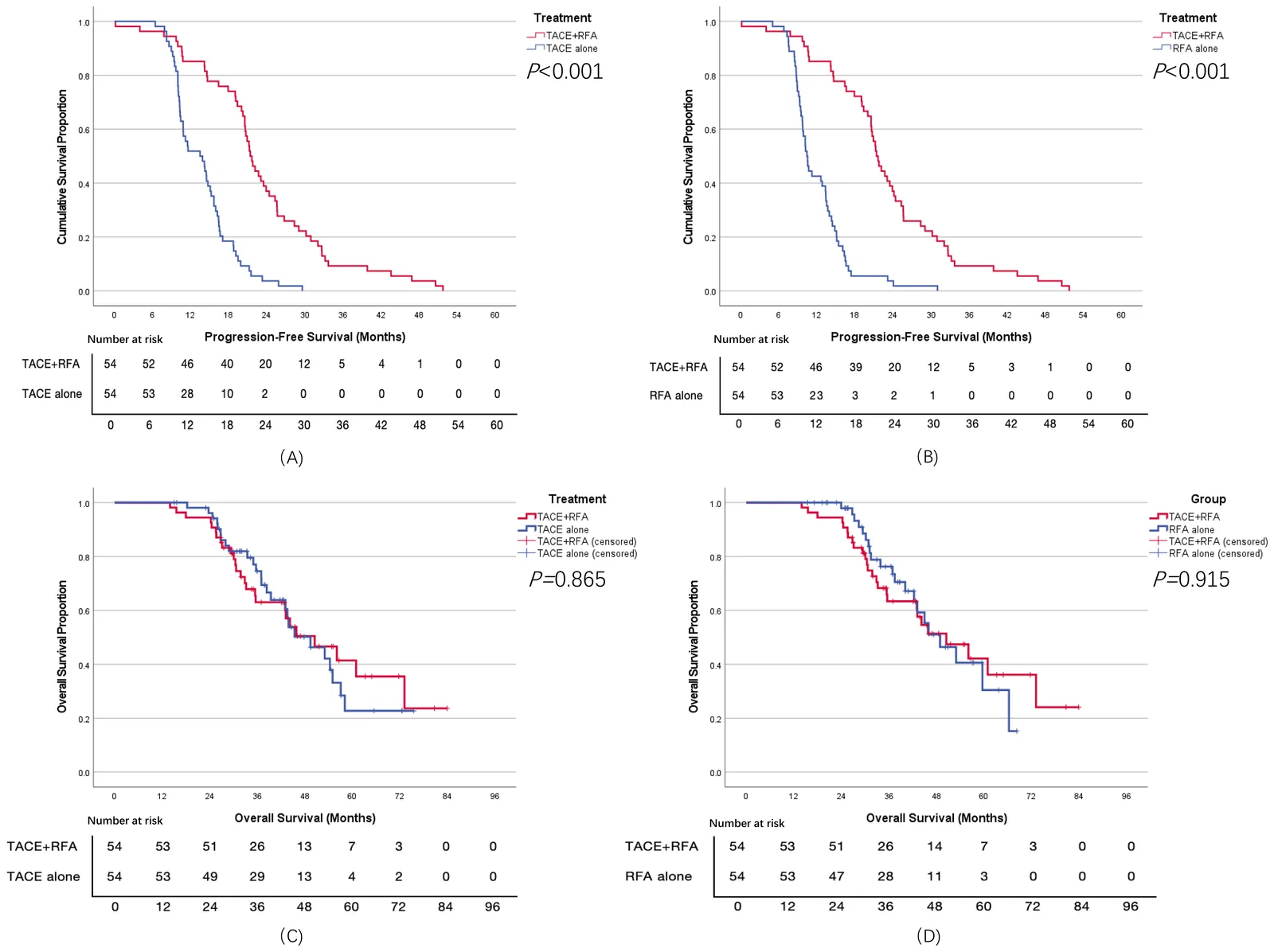
Kaplan-Meier survival curves for progression-free survival (PFS) and overall survival (OS) in the propensity score-matched cohorts. **(A)** Comparison of PFS between the TACE-RFA combination therapy and TACE monotherapy groups (P < 0.001). **(B)** Comparison of PFS between the TACE-RFA combination therapy and RFA monotherapy groups (P < 0.001). **(C)** Comparison of OS between the TACE-RFA combination therapy and TACE monotherapy groups (P = 0.865). **(D)** Comparison of OS between the TACE-RFA combination therapy and RFA monotherapy groups (P = 0.915). P values were calculated using the log-rank test. The number of patients at risk is provided below each corresponding graph. PFS, progression-free survival; OS, overall survival; TACE, transarterial chemoembolization; RFA, radiofrequency ablation.

The Kaplan-Meier survival analysis revealed significant differences in progression-free survival (PFS) among the matched cohorts. In the first cohort comparing the combination therapy with TACE monotherapy, the median PFS for the TACE-RFA group was 21.4 months (95% CI: 19.9–22.9), which was significantly longer than the 13.5 months (95% CI: 9.8–17.1) observed in the TACE alone group (P < 0.001) (**Figure 2A**). Consistent with this finding, in the second matched cohort, the combination therapy also demonstrated a superior median PFS of 21.4 months (95% CI: 19.9–22.9) compared to 10.4 months (95% CI: 9.5–11.3) in the RFA monotherapy group (P < 0.001) (**Figure 2B**). (Note: The identical median survival estimates and 95% CIs reported for the TACE-RFA group across these two separate comparisons—as well as in the subsequent subgroup analyses—result from a 96.3% [52/54] patient overlap in the respective matched combination subsets, which leads to mathematically identical Kaplan-Meier estimates).

#### 3.2.2 Overall survival

Regarding overall survival (OS), the combination group exhibited comparable outcomes to both monotherapy groups. In the comparison with TACE alone, the median OS was 50.5 months (95% CI: 37.1–63.9) for the combination therapy versus 49.4 months (95% CI: 38.8–60.0) for the TACE alone group (P = 0.865) (**Figure 2C**). Similarly, when compared with RFA monotherapy, the median OS reached 50.5 months (95% CI: 37.1–63.9), which was not significantly different from the 48.9 months (95% CI: 38.9–58.9) observed in the RFA group (P = 0.915) (**Figure 2D**).

#### 3.2.3 Subgroup analysis by tumor size

**Figure 3:**
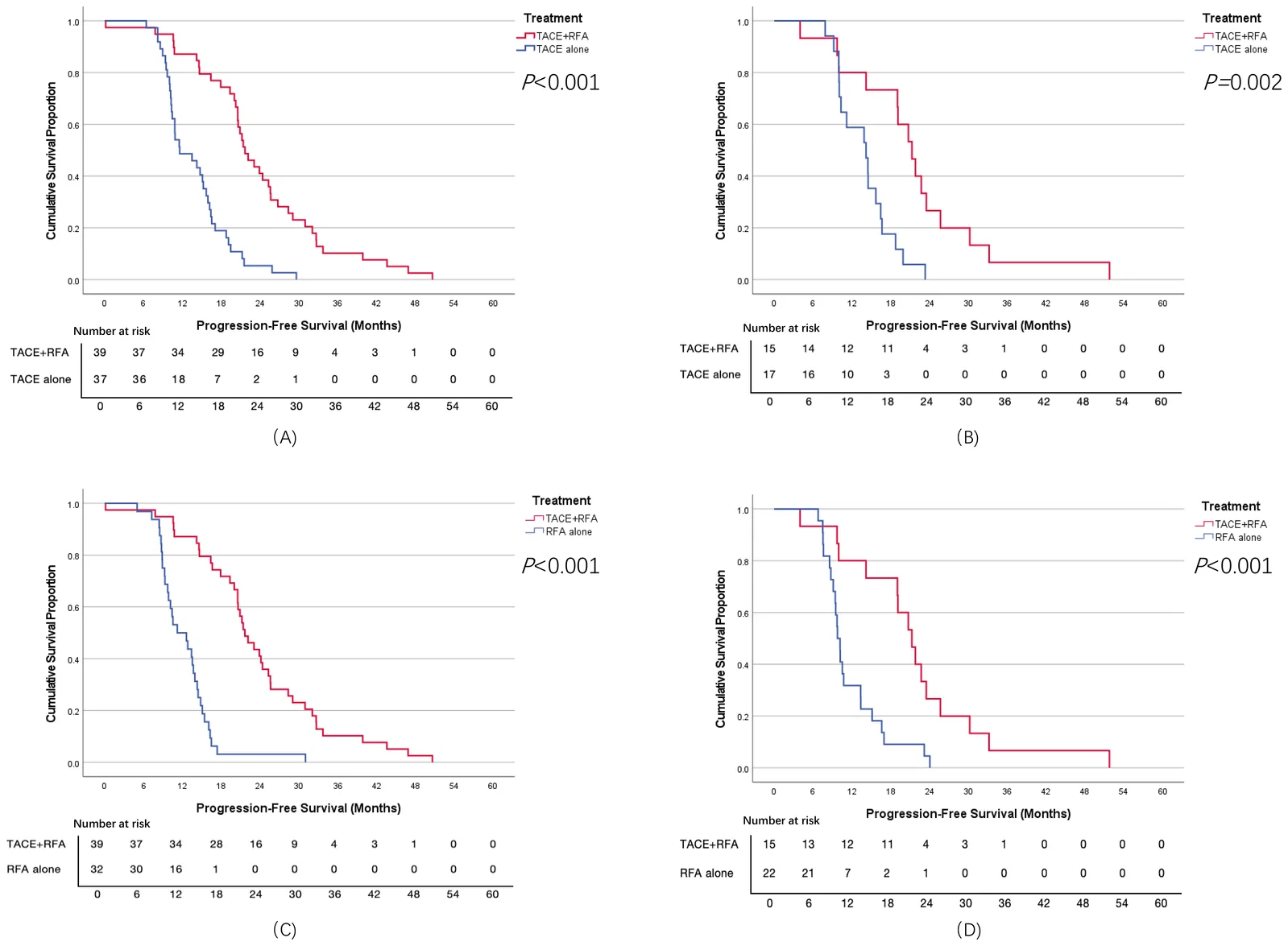
Subgroup analysis of progression-free survival (PFS) stratified by tumor size in the propensity score-matched cohorts. **(A)** Comparison of PFS between the TACE-RFA combination and TACE monotherapy in patients with a tumor size of 3–5 cm (P < 0.001). **(B)** Comparison of PFS between the TACE-RFA combination and TACE monotherapy in patients with a tumor size of 5.1–7 cm (P = 0.002). **(C)** Comparison of PFS between the TACE-RFA combination and RFA monotherapy in patients with a tumor size of 3–5 cm (P < 0.001). **(D)** Comparison of PFS between the TACE-RFA combination and RFA monotherapy in patients with a tumor size of 5.1–7 cm (P < 0.001). P values were calculated using the log-rank test. The number of patients at risk is provided below each corresponding graph. PFS, progression-free survival; TACE, transarterial chemoembolization; RFA, radiofrequency ablation.

Because the primary analysis revealed no significant differences in overall survival (OS), our subgroup analyses were focused exclusively on progression-free survival (PFS). We conducted a stratified analysis based on tumor size categories (3.0–5.0 cm and 5.1–7.0 cm). For tumors measuring 3.0–5.0 cm, the TACE-RFA combination yielded a median PFS of 21.7 months, significantly outperforming TACE alone (11.6 months, P < 0.001, **Figure 3A**) and RFA alone (11.2 months, P < 0.001, **Figure 3C**). Similarly, in the more challenging 5.1–7.0 cm subgroup, the combination therapy (median PFS: 21.3 months) maintained a distinct advantage over TACE alone (14.2 months, P = 0.002, **Figure 3B**) and RFA alone (9.8 months, P < 0.001, **Figure 3D**).

#### 3.2.4 Patterns of disease progression

We further analyzed the specific patterns of initial disease progression to evaluate the local control efficacy of the respective treatments. In the TACE-RFA versus TACE alone matched cohort, the combination therapy demonstrated a favorable trend toward reducing local tumor progression (LTP). Specifically, LTP occurred in 27.8% (15/54) of patients in the TACE-RFA group, compared to 40.7% (22/54) in the TACE alone group, although this numerical reduction did not reach statistical significance (P = 0.156).

In the TACE-RFA versus RFA alone matched cohort, the rates of LTP were comparable between the two groups (29.6% [16/54] vs. 33.3% [18/54], respectively; P = 0.679). Consequently, the corresponding rates of distant progression (including new intrahepatic lesions and extrahepatic metastasis) were 72.2% vs. 59.3% in the TACE comparison, and 70.4% vs. 66.7% in the RFA comparison.

### 3.3 Prognostic factors for progression-free survival and adjusted sensitivity analyses

To identify independent prognostic factors for PFS and to mitigate the potential impact of residual baseline imbalances observed after propensity score matching (indicated by a Standardized Mean Difference [SMD] > 0.20, as detailed in **Supplementary Tables 1**, **S2**), doubly robust estimation was systematically performed using multivariate Cox proportional hazards regression models. Crucially, to prevent model overfitting (adhering to the event-per-variable ratio) and to avoid multicollinearity (such as the overlap between BCLC and CNLC staging), a targeted inclusion strategy was employed. Variables demonstrating significance in the univariate analysis (P < 0.05), alongside clinically critical covariates with prominent residual imbalance (specifically CNLC stage, tumor size, ALB, and PT), were meticulously prioritized for the multivariate models.

In the matched cohort of the combination therapy versus TACE monotherapy, the model incorporated treatment modality, sex, ALB, PT, and AST. The analysis identified the TACE alone treatment (HR = 3.75, 95% CI: 2.41–5.85, P < 0.001) and sex (HR = 0.46, 95% CI: 0.25–0.84, P = 0.012) as independent risk factors for tumor progression (**Table 3**). Minor residual fluctuations in baseline liver function markers did not independently influence PFS.

**Table 3:** Univariate and multivariate Cox proportional hazards regression analyses for progression-free survival in the matched cohort (Combination vs. TACE alone).

Variables	Univariate analysis		Multivariate analysis	
	HR (95% CI)	*P* value	HR (95% CI)	*P* value
**Treatment (TACE alone vs Combined)**	3.84 (2.48–5.95)	<0.001*	3.75 (2.41–5.85)	<0.001*
**Sex (Female vs Male)**	0.38 (0.21–0.69)	0.001*	0.46 (0.25–0.84))	0.012*
**Age (years)**	1.01 (0.99–1.02)	0.545		
**Antiviral Therapy**	0.68 (0.32–1.48)	0.331		
**Etiology**	1.20 (0.49–2.95)	0.697		
**Child-Pugh class**	1.41 (0.71–2.81)	0.327		
**ALT (U/L)**	1.00 (0.98–1.02)	0.891		
**AST (U/L)**	0.99 (0.97–1.02)	0.530	1.01 (0.98–1.03)	0.646
**TBIL (μmol/L)**	0.99 (0.97–1.01)	0.433		
**ALB (g/L)**	1.00 (0.96–1.03)	0.785	1.01 (0.97–1.04)	0.769
**PT (sec)**	1.03 (0.91–1.18)	0.616	1.06 (0.92–1.22)	0.405
**INR**	0.89 (0.15–5.45)	0.903		
**PLT (10^9^/L)**	1.00 (0.99–1.00)	0.164		
**AFP (ng/mL)**	1.00 (1.00–1.00)	0.631		
**PIVKA-II (mAU/mL)**	1.00 (1.00–1.00)	0.434		
**Recurrence-free interval(months)**	0.99 (0.97–1.01)	0.175		
**Tumor Size (cm)**	1.08 (0.90–1.29)	0.438		
**Tumor Number**	0.89 (0.71–1.11)	0.081		
1	Reference			
2 (vs 1)	1.33 (0.85–2.09)	0.215		
3 (vs 1)	0.73 (0.44–1.18)	0.198		
**Exact Child-Pugh score**		0.080		
Score 5	Reference			
Score 6 (vs 5)	1.79 (1.03–3.12)	0.040		
Score 7 (vs 5)	1.55 (0.77–3.11)	0.219		
**ALBI grade**		0.923		
Grade 1	Reference			
Grade 2 (vs 1)	1.08 (0.72–1.60)	0.716		
Grade 3 (vs 1)	1.22 (0.17–8.84)	0.846		
**BCLC stage**	0.95 (0.64–1.39)	0.780		
CNLC stage		0.177		
Stage Ia	Reference			
Stage Ib (vs Ia)	1.73 (0.96–3.14)	0.070		
Stage IIa (vs Ib)	1.10 (0.71–1.70)	0.658		

HR, hazard ratio; CI, confidence interval; TACE, transarterial chemoembolization; RFA, radiofrequency ablation; ALT, alanine aminotransferase; AST, aspartate aminotransferase; TBIL, total bilirubin; ALB, albumin; PT, prothrombin time; INR, international normalized ratio; PLT, platelet count; AFP, alpha-fetoprotein; PIVKA-II, protein induced by vitamin K absence or antagonist-II; ALBI, albumin-bilirubin; BCLC, Barcelona Clinic Liver Cancer; CNLC, China liver cancer staging. *P < 0.05 indicates statistical significance. Bold text in the Variables column is used to distinguish main variables from their sub-categories. Bold values in the data columns indicate statistical significance (P < 0.05).

In the second matched cohort (Combination vs. RFA alone), the multivariate model incorporated treatment modality, sex, recurrence-free interval, CNLC stage, tumor size, and PT. Despite adjusting for the baseline staging disparity (which initially favored earlier stages in the combination group), the CNLC stage itself was not an independent predictor of PFS within this specific systemic-therapy background (P = 0.690). Instead, RFA monotherapy emerged as the sole independent prognostic factor associated with a significantly increased risk of tumor progression (Adjusted HR = 2.22, 95% CI: 1.71–2.87, P < 0.001) (**Table 4**).

**Table 4:** Univariate and multivariate Cox proportional hazards regression analyses for progression-free survival in the matched cohort (Combination vs. RFA alone).

Variables	Univariate analysis		Multivariate analysis	
	HR (95% CI)	*P* value	HR (95% CI)	*P* value
**Treatment (RFA alone vs Combined)**	2.15 (1.72–2.68)	<0.001*	2.22 (1.71–2.87)	<0.001*
**Sex (Female vs Male)**	0.57 (0.34–0.97)	0.037*	1.18 (0.66–2.13)	0.575
**Age (years)**	1.01 (0.99–1.02)	0.525		
**Antiviral Therapy**	0.80 (0.41–1.60)	0.532		
**Etiology**	1.10 (0.51–2.38)	0.805		
**Child-Pugh class**	2.01 (0.87–4.63)	0.103		
**ALT (U/L)**	0.99 (0.97–1.01)	0.489		
**AST (U/L)**	0.99 (0.97–1.02)	0.503		
**TBIL (μmol/L)**	1.00 (0.97–1.02)	0.824		
**ALB (g/L)**	1.00 (0.97–1.03)	0.990		
**PT (sec)**	1.05 (0.92–1.19)	0.502	1.13 (0.97–1.30)	0.108
**INR**	2.02 (0.34–11.96)	0.439		
**PLT (10^9^/L)**	1.00 (1.00–1.00)	0.594		
**AFP (ng/mL)**	1.00 (1.00–1.00)	0.648		
**PIVKA-II (mAU/mL)**	1.00 (1.00–1.00)	0.673		
**Recurrence-free interval(months)**	0.98 (0.96–1.00)	0.015*	0.99 (0.97–1.01)	0.254
**Tumor Size (cm)**	1.13 (0.95–1.34)	0.161	1.07 (0.86–1.33)	0.563
**Tumor Number**		0.616		
1	Reference			
2 (vs 1)	1.15 (0.72–1.82)	0.561		
3 (vs 1)	0.88 (0.55–1.42)	0.599		
**Exact Child-Pugh score**		0.140		
Score 5	Reference			
Score 6 (vs 5)	1.40 (0.79–2.50)	0.250		
Score 7 (vs 5)	2.12 (0.91–4.92)	0.081		
**ALBI grade**		0.666		
Grade 1	Reference			
Grade 2 (vs 1)	1.00 (0.66–1.51)	0.997		
Grade 3 (vs 1)	1.91 (0.47–7.87)	0.369		
**BCLC stage**	1.02 (0.69–1.50)	0.934		
**CNLC stage**		0.278		0.690
Stage Ia	Reference		Reference	
Stage Ib (vs Ia)	1.60 (0.90–2.86)	0.110	0.80 (0.38–1.69)	0.559
Stage IIa (vs Ib)	1.20 (0.76–1.88)	0.435	0.80 (0.49–1.33)	0.392

HR, hazard ratio; CI, confidence interval; TACE, transarterial chemoembolization; RFA, radiofrequency ablation; ALT, alanine aminotransferase; AST, aspartate aminotransferase; TBIL, total bilirubin; ALB, albumin; PT, prothrombin time; INR, international normalized ratio; PLT, platelet count; AFP, alpha-fetoprotein; PIVKA-II, protein induced by vitamin K absence or antagonist-II; ALBI, albumin-bilirubin; BCLC, Barcelona Clinic Liver Cancer; CNLC, China liver cancer staging. *P < 0.05 indicates statistical significance. Bold text in the Variables column is used to distinguish main variables from their sub-categories. Bold values in the data columns indicate statistical significance (P < 0.05).

Collectively, these analyses confirm that the profound survival benefit conferred by the sequential TACE-RFA strategy is intrinsically driven by treatment synergism, rather than being confounded by residual baseline variations.

### 3.4 Safety and health economic outcomes

The perioperative safety and health economic indicators were comprehensively evaluated across the matched cohorts (**Table 5**).

**Table 5:** Comparison of post-procedural pain, length of hospital stay, and total costs across the propensity score-matched cohorts.

Variables	Cohort 1: vs. TACE alone			Cohort 2: vs. RFA alone		
	TACE+RFA (n=54)	TACE (n=54)	*P* value	TACE+RFA (n=54)	RFA (n=54)	*P* value
Significant post-procedural pain, n (%)	22 (40.7%)	21 (38.9%)	0.844	21 (38.9%)	15 (27.8%)	0.221
Length of hospital stay (days), median (IQR)	11.5 (9.0–14.3)	7.0 (3.0–11.0)	<0.001*	12.0 (9.0–15.0)	8.0 (6.0–12.0)	<0.001*
Total cost (CNY), median (IQR)	30, 974 (27, 733–37, 473)	17, 310 (13, 259–31, 003)	<0.001*	32, 201 (27, 788–38, 107)	16, 735 (14, 538–21, 347)	<0.001*

TACE, transarterial chemoembolization; RFA, radiofrequency ablation; IQR, interquartile range; CNY, Chinese Yuan. Categorical variables were compared using the Chi-square test, and continuous variables were compared using the Mann-Whitney U test. *P < 0.05 indicates statistical significance.

Regarding tolerability, there was no significant difference in the incidence of significant post-procedural pain between the TACE-RFA combination group and the TACE alone group (40.7% vs. 38.9%, P = 0.844), nor between the combination group and the RFA alone group (38.9% vs. 27.8%, P = 0.221). This indicates that the addition of sequential treatments did not significantly increase patient discomfort.

As anticipated, due to the dual nature of the procedure during a single admission, the combination therapy was inherently associated with longer hospital stays and higher costs. Compared to TACE monotherapy, the combination group had a significantly longer median length of hospital stay (11.5 vs. 7.0 days, P < 0.001) and higher median hospital costs (30, 974 vs. 17, 310 CNY, P < 0.001). Similar trends were observed when compared to RFA monotherapy (Length of stay: 12.0 vs. 8.0 days, P < 0.001; Costs: 32, 201 vs. 16, 735 CNY, P < 0.001). Nevertheless, considering the substantial prolongation of progression-free survival, these initial perioperative investments represent a potentially cost-effective trade-off for long-term tumor control.

### 3.5 Representative case illustrations

**Figure 4:**
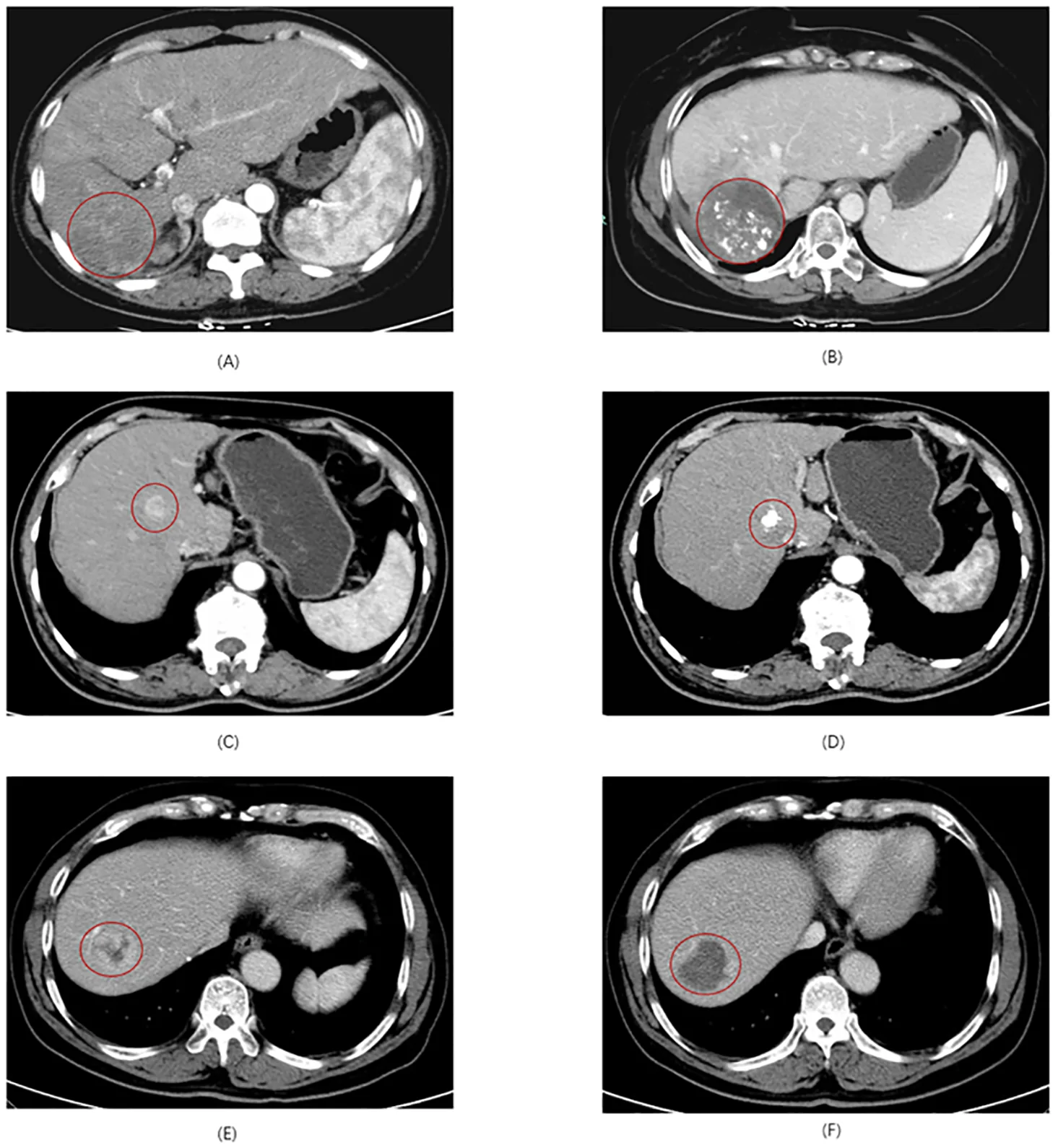
Representative contrast-enhanced computed tomography CT images of the three locoregional treatment modalities for recurrent hepatocellular carcinoma. The target recurrent lesions are indicated by red circles. **(A, B)** A patient from the TACE-RFA combination group. **(A)** Pre-treatment arterial phase CT reveals a recurrent tumor in the right hepatic lobe. **(B)** Follow-up CT after sequential TACE-RFA therapy demonstrates an adequate, non-enhancing ablation zone encompassing the original lesion, accompanied by dense lipiodol deposition. **(C, D)** A patient from the TACE monotherapy group. **(C)** Pre-treatment CT displays a recurrent nodule. **(D)** Post-treatment CT illustrates lipiodol retention within the target lesion following chemoembolization. **(E, F)** A patient from the RFA monotherapy group. **(E)** Pre-treatment CT shows a recurrent tumor. **(F)** Post-treatment CT demonstrates a well-defined ablation defect covering the initial tumor area. CT, computed tomography; TACE, transarterial chemoembolization; RFA, radiofrequency ablation.

To visually illustrate the typical radiological features and morphological changes associated with the different interventions, representative imaging cases before and after treatments are presented in **Figure 4**.

## 4 Discussion

To the best of our knowledge, this study provides one of the most rigorously matched real-world comparisons evaluating the efficacy, safety, and resource consumption of the TACE-RFA sequential combination therapy against either TACE or RFA monotherapy for recurrent hepatocellular carcinoma (rHCC), within the contemporary context of a uniform systemic therapy background (Lenvatinib plus PD-1 inhibitors). Through strict propensity score matching, we substantially reduced measured baseline imbalances, thereby improving the comparability between treatment groups. Our principal findings demonstrate that the TACE-RFA combination strategy significantly prolongs progression-free survival (PFS) and reduces the risk of tumor progression compared to both monotherapies, without compromising patient safety or increasing post-procedural pain, despite the anticipated increases in initial hospital stay and costs.

The most striking finding of this study is the profound PFS advantage conferred by the combination therapy. The median PFS in the TACE-RFA group reached an impressive 21.4 months, which was approximately 8 months longer than that of the matched TACE cohort (13.5 months) and 11 months longer than the matched RFA cohort (10.4 months) in their respective independent analyses. Multivariate Cox regression further cemented TACE-RFA as an independent protective factor against tumor recurrence. This superior local tumor control is biologically plausible and can be attributed to a multifaceted synergistic interaction between the two modalities. First, TACE serves to severely restrict arterial inflow, which theoretically diminishes the cooling effect of hepatic blood flow (the “heat-sink effect”). However, considering that the interval between TACE and RFA could extend up to 4 weeks in this cohort, the persistence of complete arterial stasis at the exact time of RFA may be variable, despite our utilization of supplemental embolic agents (such as blank microspheres or gelatin sponge). Therefore, rather than a permanent eradication of the heat-sink effect, the synergistic advantage is more likely a combination of prolonged ischemia-induced cellular hypoxia—rendering tumor cells substantially more susceptible to subsequent thermal injury—and the transient reduction of local microcirculation (18). Consequently, it becomes technically more feasible to achieve the critical 0.5 to 1.0 cm ablative safety margin, which is notoriously difficult for intermediate-sized (3.0–7.0 cm) recurrent tumors treated by RFA alone. Second, a hyperthermia-enhanced pharmacokinetic synergy occurs: the sub-lethal hyperthermia generated at the periphery of the RFA zone increases the permeability of tumor cell membranes and dilates the microvessels, thereby significantly enhancing the intracellular uptake and cytotoxic efficacy of the chemotherapeutic agents previously deposited by TACE (19). Finally, the lipiodol emulsion retained within the tumor and its satellite nodules alters the local thermal conductivity to amplify ablative efficiency, while concurrently acting as an excellent imaging marker (20). This allows for precise targeting of micro-metastases that might otherwise be invisible on standard ultrasound, thereby effectively minimizing the risk of marginal recurrence.

The magnitude of the PFS benefit observed in our cohort is broadly consistent with quantitative outcomes reported for TACE-ablation strategies in previous studies. In a study specifically involving recurrent HCC after curative resection, Zheng et al. reported a median PFS of 24 months (95% CI, 15.2–32.8 months) following TACE-RFA, which is comparable to the 21.4 months observed in our TACE-RFA cohort (21). More recently, the phase III TORCH randomized trial further demonstrated that TACE followed by thermal ablation significantly prolonged median PFS compared with TACE alone (17.7 vs. 7.3 months; HR, 0.47; 95% CI, 0.34–0.65), providing higher-level evidence for the survival benefit of the sequential TACE-ablation strategy (22). Although differences in tumor stage, recurrence status, systemic treatment, and definitions of progression preclude direct cross-study comparisons, these quantitative data collectively support the favorable disease control observed in our study.

Interestingly, despite the substantial improvement in PFS, we observed comparable overall survival (OS) across all groups. This observation must be interpreted within the context of the modern systemic therapy era. Recently, landmark phase III trials, such as EMERALD-1 (Durvalumab plus Bevacizumab with TACE) and LEAP-012 (Lenvatinib plus Pembrolizumab with TACE), have revolutionized the treatment landscape by demonstrating significant PFS benefits when combining locoregional therapies with potent systemic agents (23, 24). In our cohort, a standardized, potent systemic backbone (Lenvatinib and Tislelizumab) was uniformly administered. This potent systemic therapy significantly suppresses intrahepatic micrometastases and extrahepatic spread, drastically prolonging the baseline survival for all patients. Consequently, this universal systemic benefit likely diluted the initial OS advantage provided by the enhanced local control of TACE-RFA. Furthermore, as is common in contemporary oncology trials, patients who experienced disease progression subsequently received highly heterogeneous salvage treatments (e.g., switching to second-line targeted agents or alternative immunotherapies). This variability in post-progression care inevitably introduces survival confounding, further masking any potential OS difference. Nevertheless, the fact that TACE-RFA almost doubled the PFS—even on a background of state-of-the-art targeted and immune therapies—highlights the indispensable value of aggressive local tumor eradication.

While conventional TACE and RFA represent classical modalities, the interventional armamentarium for rHCC continues to evolve. Contemporary techniques such as Drug-Eluting Bead TACE (DEB-TACE) offer more sustained drug release and potentially less systemic toxicity, though its definitive survival advantage over conventional TACE in the recurrent setting remains under investigation (25). Similarly, transarterial radioembolization (TARE), also referred to as selective internal radiation therapy (SIRT), using Yttrium-90 has emerged as an important alternative locoregional approach capable of achieving durable tumor control. In the multicenter LEGACY study, which enrolled patients with solitary unresectable HCC measuring up to 8 cm, Yttrium-90 radioembolization achieved an objective response rate of 88.3%, with 62.2% of patients maintaining a response for at least 6 months; the 3-year overall survival rate was 86.6% (26). These findings are particularly relevant to the 3–7 cm tumor-size range evaluated in our study and suggest that SIRT represents a credible alternative to TACE-ablation when technically feasible and appropriately selected. More recently, a direct comparison of Yttrium-90 radiation segmentectomy and combined TACE plus thermal ablation for HCCs measuring up to 5 cm demonstrated comparable pathologic tumor necrosis (83.8% vs. 79.0%) and complete pathologic necrosis rates (61.5% vs. 45.0%, respectively), further supporting the potential of radioembolization to achieve local tumor control comparable to combined embolization-ablation strategies in selected patients (27). Nevertheless, because these radioembolization studies predominantly involved treatment-naïve or transplant-eligible HCC rather than recurrent HCC treated against a uniform systemic therapy background, direct extrapolation to our population should be made cautiously. Prospective head-to-head studies comparing TACE-ablation with SIRT specifically in patients with recurrent HCC measuring 3–7 cm are therefore warranted. Furthermore, Microwave Ablation (MWA) is increasingly favored over RFA for tumors >3 cm due to its faster heating mechanisms and reduced susceptibility to the heat-sink effect (28). Future studies exploring the combination of these advanced locoregional technologies (e.g., DEB-TACE plus MWA) alongside novel immunotherapies are highly anticipated to further optimize rHCC management.

From a safety and health economics perspective, our study addresses a critical clinical concern: does combining two invasive procedures expose patients to unacceptable risks? Our data provides a reassuringly negative answer. The incidence of significant post-procedural pain was comparable between the combination and monotherapy groups (approximately 38.9%-40.7%), confirming that the addition of RFA to TACE is highly tolerable. Predictably, the sequential combination therapy necessitated a longer median hospital stay (11.5-12.0 days) and incurred higher total procedural costs (over 30, 000 CNY) compared to standalone treatments. However, in the context of value-based oncology, these initial perioperative investments must be weighed against the long-term clinical dividends. By almost doubling the median PFS, the combination therapy effectively delays the psychological and physical burden of tumor recurrence, potentially reducing the frequency and costs of subsequent salvage hospitalizations.

This study has several limitations that warrant acknowledgment. First, its retrospective, single-center design makes it susceptible to inherent selection biases, although we employed rigorous PSM to mitigate this issue. However, due to the strict matching criteria, the final sample size in each sub-cohort was relatively small (54 pairs each), which might have limited the statistical power to detect subtle differences in long-term OS. Second, it should be emphasized that this study was specifically designed to evaluate the combination therapy against monotherapies through two distinct matching procedures; therefore, the current matched populations do not permit a direct statistical comparison of efficacy between the TACE alone and RFA alone groups. Third, owing to limitations in historical medical records, a comprehensive grading of all adverse events according to the Common Terminology Criteria for Adverse Events (CTCAE) criteria was not feasible, prompting us to focus on the incidence of significant pain as a surrogate for tolerability. Fourth, while the first-line systemic background therapy was rigorously standardized prior to progression, the subsequent salvage treatments (such as switching targeted agents or immunotherapies) administered after disease progression could not be controlled. This variability in post-progression care inevitably acts as a confounding factor, which likely influenced the overall survival outcomes and explains the discordant PFS and OS results. Fifth, although rigorous PSM was employed, an assessment of SMDs revealed residual imbalances, particularly a higher proportion of CNLC stage Ia patients in the combination group compared to the RFA monotherapy group. Potentially, this residual imbalance could have conferred a baseline prognostic advantage to the combination cohort, artificially inflating its apparent efficacy. Nevertheless, our subsequent doubly robust sensitivity analysis—which explicitly adjusted for these imbalanced baseline covariates—demonstrated that the TACE-RFA regimen remained a strong, independent protective factor. Thus, the observed survival benefit is largely attributable to the synergistic treatment effect rather than baseline confounding. Finally, although explicit clinical staging (such as BCLC or CNLC classifications) was not directly utilized as a covariate in the initial propensity score model, no statistically significant between-group differences in stage distribution were observed after matching. Nevertheless, some residual imbalance cannot be completely excluded. Coupled with our rigorously defined inclusion criteria (including a strict ECOG PS of 0 and well-compensated liver function), this ensures the robust comparability and international generalizability of our matched cohorts.

## 5 Conclusion

In conclusion, for patients with recurrent hepatocellular carcinoma treated on a uniform background of systemic therapy (Lenvatinib plus PD-1 inhibitors), the sequential TACE-RFA combination strategy yields a significantly superior progression-free survival compared to TACE or RFA monotherapy. While this combined approach inherently requires a longer initial hospital stay and higher procedural costs, it does not increase patient discomfort or complication rates. Given its robust local tumor control, TACE-RFA represents a highly effective and clinically justified locoregional strategy for managing rHCC in the modern era of combined therapies. Future large-scale, prospective randomized controlled trials are needed to validate these findings across various systemic treatment regimens.

## Data Availability

The raw data supporting the conclusions of this article will be made available by the authors, without undue reservation.
