# Supplementary material for: Comparative evaluation of TACE-RFA versus monotherapies on a background of systemic therapy for recurrent hepatocellular carcinoma: a propensity score-matched analysis
Source: Front Oncol. 2026 Aug 26;16:1825546. doi: 10.3389/fonc.2026.1825546 (PMC13553491; doi:10.3389/fonc.2026.1825546)
Supplement: Supplementary file 1 [file Table1.docx]

Table S1. Standardized mean differences of baseline covariates before and after propensity score matching (Combination vs. TACE alone)

| Variables | Before PSM | After PSM |
| --- | --- | --- |
|  | SMD | SMD |
| **Age (years)** | 0.085 | 0.015 |
| **Sex (Male/Female)** | 0.05 | 0 |
| **ALT(U/L)** | 0.177 | 0.010 |
| **AST (U/L)** | 0.176 | 0.278 |
| **TBIL (μmol/L)** | 0.179 | 0.138 |
| **ALB (g/L)** | 0.493 | 0.349 |
| **PT (sec)** | 0.311 | 0.361 |
| **INR** | 0.047 | 0.154 |
| **PLT (10^9^/L)** | 0.311 | 0.018 |
| **AFP (ng/mL)** | 0.021 | 0.046 |
| **PIVKA-II (mAU/mL)** | 0.043 | 0.051 |
| **Tumor Size (cm)** | 0.597 | 0.062 |
| **Etiology (HBV/Others)** | 0.186 | 0.09 |
| **Antiviral Therapy (Yes/No)** | 0.004 | 0.07 |
| **Tumor Number** |  |  |
| 1 | 0.468 | 0.036 |
| 2 | 0.387 | 0.163 |
| 3 | 0.085 | 0.124 |
| **Child-Pugh class (A/B)** | 0.04 | 0.069 |
| **Exact Child-Pugh score (A5/A6/B7)** |  |  |
| A5 | 0.301 | 0.222 |
| A6 | 0.371 | 0.210 |
| A7 | 0.04 | 0.069 |
| **ALBI grade (1/2/3)** |  |  |
| 1 | 0.349 | 0.154 |
| 2 | 0.389 | 0.192 |
| 3 | 0.191 | 0.197 |
| **BCLC stage (A/B)** | 0.503 | 0.074 |
| **CNLC stage (Ia/Ib/IIa)** |  |  |
| Ia | 0.519 | 0.120 |
| Ib | 0.059 | 0.052 |
| IIa | 0.503 | 0.074 |
| **Recurrence-free interval (months)** | 0.055 | 0.111 |

Abbreviations: SMD, standardized mean difference; PSM, propensity score matching; ALT, alanine aminotransferase; AST, aspartate aminotransferase; TBIL, total bilirubin; ALB, albumin; PT, prothrombin time; INR, international normalized ratio; PLT, platelet count; AFP, alpha-fetoprotein; PIVKA-II, protein induced by vitamin K absence or antagonist-II; HBV, hepatitis B virus; ALBI, albumin-bilirubin; BCLC, Barcelona Clinic Liver Cancer; CNLC, China Liver Cancer.

Table S2. Standardized mean differences of baseline covariates before and after propensity score matching (Combination vs. RFA alone)

| Variables | Before PSM | After PSM |
| --- | --- | --- |
|  | SMD | SMD |
| **Age (years)** | 0.229 | 0.201 |
| **Sex (Male/Female)** | 0.265 | 0.099 |
| **ALT(U/L)** | 0.318 | 0.148 |
| **AST (U/L)** | 0.423 | 0.208 |
| **TBIL (μmol/L)** | 0.220 | 0.117 |
| **ALB (g/L)** | 0.230 | 0.243 |
| **PT (sec)** | 0.320 | 0.350 |
| **INR** | 0.079 | 0.043 |
| **PLT (10^9^/L)** | 0.099 | 0.152 |
| **AFP (ng/mL)** | 0.116 | 0.051 |
| **PIVKA-II (mAU/mL)** | 0.031 | 0.043 |
| **Tumor Size (cm)** | 0.524 | 0.301 |
| **Etiology (HBV/Others)** | 0.506 | 0.229 |
| **Antiviral Therapy (Yes/No)** | 0.328 | 0.069 |
| **Tumor Number** |  |  |
| 1 | 0.164 | 0.188 |
| 2 | 0.145 | 0.166 |
| 3 | 0.034 | 0.041 |
| **Child-Pugh class (A/B)** | 0.131 | 0 |
| **Exact Child-Pugh score (A5/A6/B7)** |  |  |
| A5 | 0.034 | 0.095 |
| A6 | 0.130 | 0.110 |
| A7 | 0.132 | 0 |
| **ALBI grade (1/2/3)** |  |  |
| 1 | 0.035 | 0 |
| 2 | 0.045 | 0.040 |
| 3 | 0.033 | 0.194 |
| **BCLC stage (A/B)** | 0.235 | 0.263 |
| **CNLC stage (Ia/Ib/IIa)** |  |  |
| Ia | 0.488 | 0.472 |
| Ib | 0.232 | 0.192 |
| IIa | 0.236 | 0.263 |
| **Recurrence-free interval (months)** | 0.291 | 0.285 |

Abbreviations: SMD, standardized mean difference; PSM, propensity score matching; ALT, alanine aminotransferase; AST, aspartate aminotransferase; TBIL, total bilirubin; ALB, albumin; PT, prothrombin time; INR, international normalized ratio; PLT, platelet count; AFP, alpha-fetoprotein; PIVKA-II, protein induced by vitamin K absence or antagonist-II; HBV, hepatitis B virus; ALBI, albumin-bilirubin; BCLC, Barcelona Clinic Liver Cancer; CNLC, China Liver Cancer.
